# Rescue the Failed Half-ZFN by a Sensitive Mammalian Cell-Based Luciferase Reporter System

**DOI:** 10.1371/journal.pone.0045169

**Published:** 2012-09-18

**Authors:** Weifeng Zhang, Yuanxu Guo, Chen Zhang, Haiyan Ji, Wenpeng Meng, Dongyang Wang, Xing Li, Qinwen Mao, Haibin Xia

**Affiliations:** 1 Laboratory of Gene Therapy, Department of Biochemistry, College of Life Sciences, Shaanxi Normal University, Xi’an, Shaanxi, People’s Republic of China; 2 Departmet of Pathology, Northwestern University Feinberg School of Medicine Chicago, Chicago, Illinois, United States of America; German Cancer Research Center, Germany

## Abstract

ZFN technology is a powerful research tool and has been used for genome editing in cells lines, animals and plants. The generation of functional ZFNs for particular targets in mammalian genome is still challenging for an average research group. The modular-assembly method is relatively fast, easy-to-practice but has a high failure rate. Some recent studies suggested that a ZFP with low binding activity might be able to form a working ZFN pair with another binding active half-ZFP. In order to unveil the potential ZFP candidates among those with low binding activities, this paper established a highly sensitive mammalian cell-based transcriptional reporter system to assess the DNA binding activities of ZFPs by inserting multiple copies of ZFN target sequence fragment (TSF) of an interested gene (e. g., hPGRN or hVEGF). Our results showed that this system increased the screening sensitivity up to 50-fold and markedly amplified the differences in the binding activities between different ZFPs. We also found that the targeted chromosomal gene repair efficiency of each hPGRN or hVEGF ZFN pair was in proportion with the combination of the binding activities of the ZFL (Left zinc finger) and ZFR (Right zinc finger). A hPGRN ZFR with low binding ability was able to form a biological active ZFN if combined with a hPGRN ZFL with relatively high binding ability. Lastly, site-specific genome editing by hPGRN ZFNs generated by this system was confirmed by sequencing, and the PGRN knock-out cell line showed significantly decreased cell growth compared with the control. Our system will provide a valuable tool for further optimizing the nucleases with regard to specificity and cytotoxicity.

## Introduction

Spontaneous genome modification by homologous recombination (HR) has a very low frequency (∼1 HR event per 10^6^ cells) in mammalian cells [1]. An artificially induced double strand break (DSB) of DNA can greatly increase the efficiency of HR through physiological DNA repair machinery. Zinc finger nucleases (ZFNs), which are generated by fusing a zinc finger DNA binding domain to the Fok1 DNA cleavage domain, have been successfully used to induce site-specific DSB and genome modifications in different species such as Drosophila, plants, rats, and in different cell lines such as CD4+ T cell, iPSCs, hESCs, etc [2], [3], [4], [5], [6].

The generation of highly specific engineered ZFNs is a crucial step toward the application of ZFNs in both basic research and clinical therapeutics. Three methods have been described for the production of ZFNs: a proprietary method used by Sangamo Biosciences [7], the modular-assembly method via standard recombinant DNA technology and cell-based selection methods [8], [9], [10], [11], [12], [13], [14]. The selection methods are highly labor-intensive and time-consuming. In contrast, the modular-assembly method is fast, easy-to-practice. Simply by using a computer-generated archive the researchers can build ZFNs from one- or two-finger modules [14], [15], [16], [17], [18]. However, they often face the problems that the ZFNs, either one or both halves, just fail to work [16], [19]. When both halves fail, it is probably simplest to start all over again on another target site. What if only one half of the nuclease fails? Herrmann et al. recently adapted a commercial yeast one-hybrid kit to select zinc fingers from targeted mini-libraries to rescue ‘orphan’ half-ZFNs [20]. Interestingly, some preliminary experiments in our laboratory showed that the combination of a good and suboptimal ‘failed’ half-ZFN occasionally resulted in a working ZFN pair. An analysis of off-target cutting has also suggested that a shortage of binding energy from a half-site can be energetically compensated by excess zinc finger: DNA binding energy in the other half-site [21]. Hence, we postulated that a ‘failed’ half ZFN might actually contain enough binding activity, although at a level below the detection limit of the current technique, to form a working ZFN pair with another binding active half-ZFN.

In order to prove our hypothesis, a highly sensitive mammalian cell-based transcriptional reporter system was established in an attempt to unveil the potential ZFP fingers among those with low binding activities. ZFN pairs were then generated by using ZFLs and ZFRs with different binding activities followed by assessment of gene repair efficiency. It was showed that our system was a valuable tool for further optimizing the nucleases with regard to specificity and cytotoxicity.

## Results

### Establishment of a Highly Sensitive Transcriptional Reporter System

**Figure 1:**
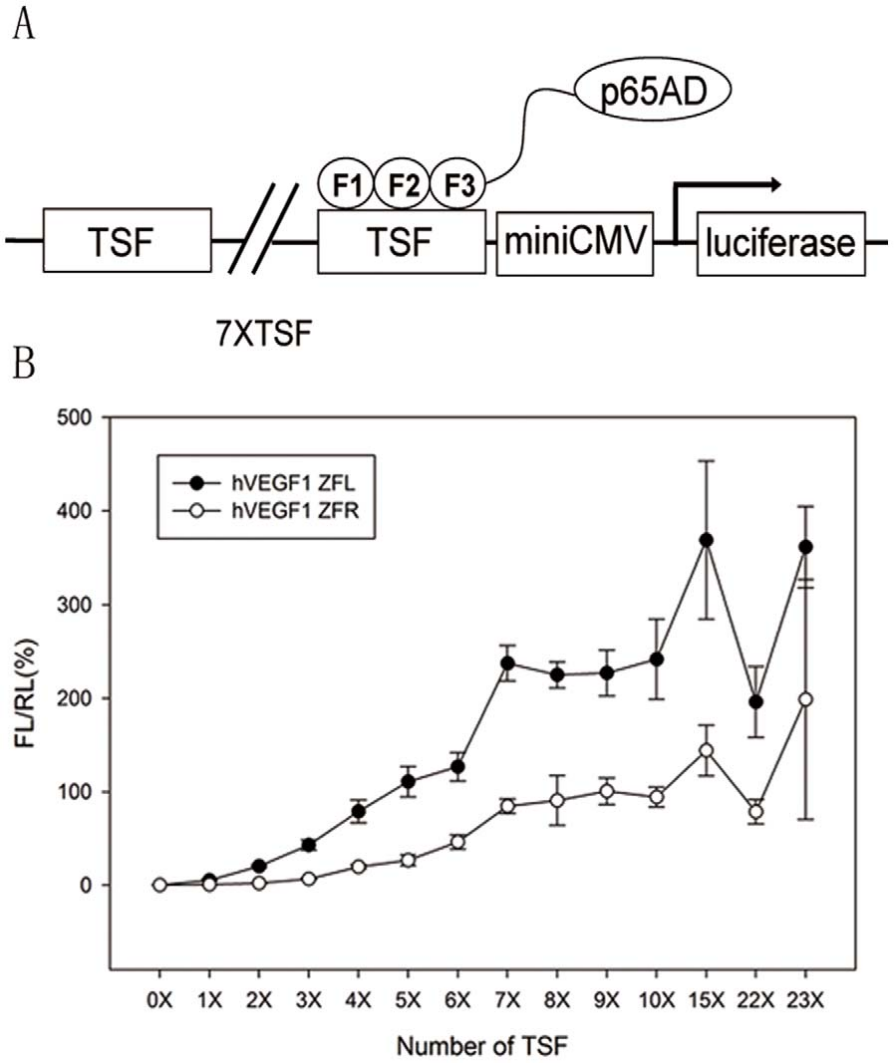
Binding capacities of assembled ZFPs assessed by the EZSS system. (A) A cartoon of the EZSS system, which is composed of a transcription factor (TF)-expressing vector and a reporter vector. The reporter vector is composed of ZFN target sequence fragment (TSF), a mini-CMV promoter and a firefly luciferase reporter gene. The transcription factor (TF)-expressing vector expresses a fusion protein consisting of p65AD and a ZFP. (B) The binding activities of hVEGF1 ZFL and hVEGF1 ZFR. Different copy numbers of TSF for hVEGF1 ZFL or ZFR were inserted upstream of the mini-CMV promoter. The two vectors of the EZSS system were co-transfected into HEK 293 cells. Forty-eight hrs post-transfection, the firefly luciferase and internal control, renilla luciferase activities were quantified. The ratio of firefly luciferase and renilla luciferase activity (FL/RL) was used to reflect the ZFP binding activities.

**Figure 2:**
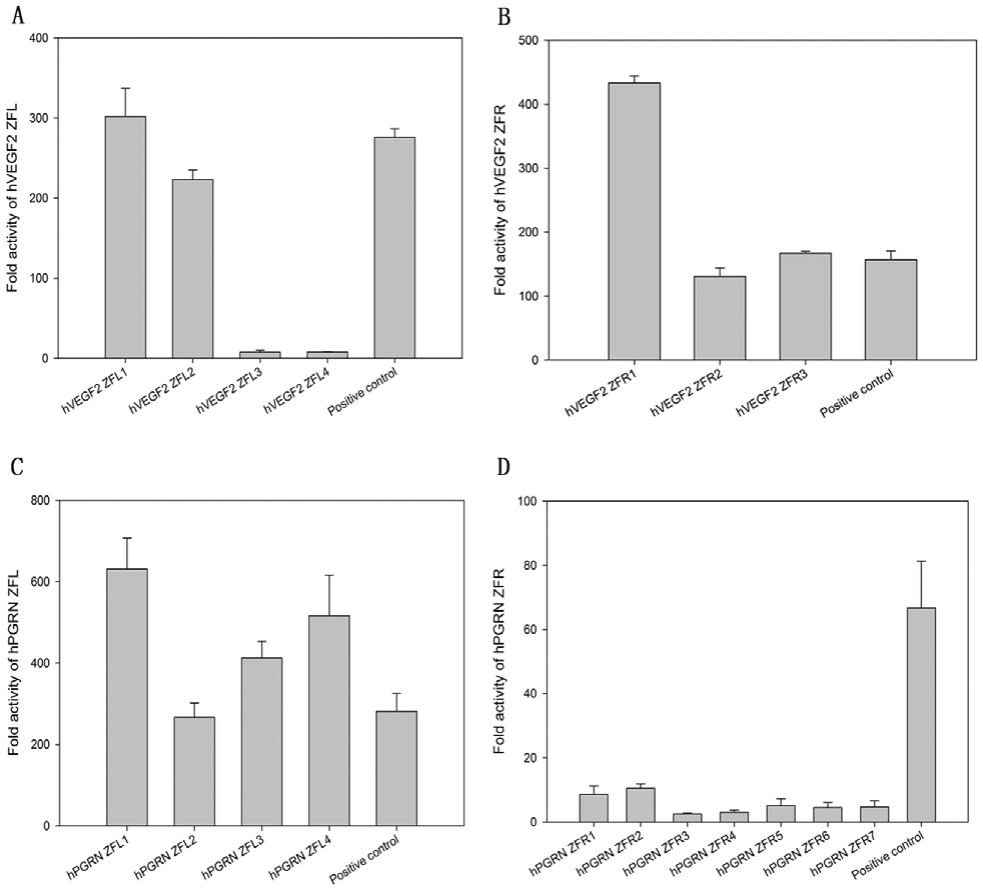
The binding activities of hVEGF2 ZFPs (A, B) and hPGRN ZFPs (C, D) determined by luciferase activities. Fold activation of transcription in the EZSS system was determined by the ratio of luciferase activity from HEK 293 cells transfected with reporter vector and the plasmid carrying a zinc finger array fused to p65 AD to that from HEK 293 cells transfected with reporter vector only. All determinations were performed in triplicate and the means and standard deviations of these means are shown.

In order to unveil the potential ZFP candidates, especially among those with low binding activities assessed by conventional transcriptional reporter system [15], [22], we established a highly sensitive mammalian cell-based luciferase reporter system (named eukaryotic zinc finger screening system, EZSS) to detect the binding activities of ZFPs. Briefly, the system consists of a transcription factor (TF)-expressing vector and a reporter vector. The former is composed of a transcription activation domain of p65 (p65 AD) fused with ZFPs, and the latter is composed of a ZFN target sequence fragment (TSF), a mini-CMV promoter (a minimal CMV promoter) [23] and a firefly luciferase reporter gene. In order to increase the detection sensitivity of the system, we inserted different numbers of copies of the interested TSF upstream of mini-CMV promoter in reporter vector (Figure 1A). For the proof-of-principle, a ZFP, which has been reported to be able to target human VEGF genome (the TSF named as hVEGF1) by Maeder et al. [15], was inserted in the TF-expressing vector system. Then the TF-expressing vector was co-transfected with the reporter vector with TSF recognized by hVEGF1 ZFP into HEK 293 cells followed by luciferase activity assay. With the increase of the copy numbers of TSF recognized by hVEGF1 ZFL or ZFR in the reporter vectors, the luciferase activities elevated almost linearly until the copy number of TSF reached 7. Much higher copies resulted in only slightly higher and much noisier expression (Figure 1B). This tendency was found in all other tested ZFPs (data available if requested). The EZSS system was then used to screen potential ZFPs targeting hVEGF or hPGRN. We synthesized 7 and 11 DNA-binding domains that recognize a single target site of hVEGF (hVEGF2, a new TSF site different from hVEGF1) (Table 1) and human progranulin (hPGRN) (Table 2) genome, respectively. DNA binding capacities of ZFPs that target hVEGF2 or hPGRN assessed by EZSS with 7 TSF repeats were illustrated in Figure 2. The relative light units (RLU) from EZSS with 1 X TSF recognized by hPGRN ZFPs ranged from 4.0 to 0.4; that from EZSS with 7 X TSF ranged from 218.2 to 0.8; the RLU ratio of EZSS with 7 X TSF to those with 1 X TSF ranged from 2.0 to 54.3 (Table 3). It appeared that EZSS was highly sensitive and markedly amplified the differences in the binding activities between different ZFPs, which would allow us to find potential effective ZFPs among those with low binding activities.

**Table 1:** The target sequences, corresponding key amino acid sequences and binding affinities of hVEGF2 zinc fingers (ZF).

ZF name	F1 subsite/RH sequence	F2 subsite/RH sequence	F3 subsite/RH sequence	Binding affinities
	GGT	GGA	GGA	
**hVEGF2 ZFL1**	IPNHLAR	QSAHLKR	QSAHLKR	302
**hVEGF2 ZFL2**	LRHHLEA	QSPHLKR	QSPHLKR	223
**hVEGF2 ZFL3**	KHSNLTR	RNENLAR	RNENLAR	8
**hVEGF2 ZFL4**	QKHHLAV	QSAHLKR	QSAHLKR	8
	GGA	GGC	GGC	
**hVEGF2 ZFR1**	EESNLRR	RPDVLAR	RPDVLAR	433
**hVEGF2 ZFR2**	EESNLRR	RRESLVR	RRESLVR	131
**hVEGF2 ZFR3**	DEANLRR	RKDDLKR	RKDDLKR	167

Binding affinities are expressed as mean fold-activation of transcription in the EZSS system.

**Table 2:** The target sequences, corresponding key amino acid sequences and binding affinities of hPGRN ZF.

ZF name	F1 subsite/RH sequence	F2 subsite/RH sequence	F3 subsite/RH sequence	Binding affinities
	GAC	GGT	GAA	
**hPGRN ZFL1**	EEANLRR	LRHHLTR	QKANLTR	631
**hPGRN ZFL2**	DRSNLTR	LRHHLTR	QKANLTR	267
**hPGRN ZFL3**	EESNLRR	LRHHLTR	QKANLTR	413
**hPGRN ZFL4**	EQSNLRR	LRHHLTR	QKANLTR	516
	GGT	GCT	TGG	
**hPGRN ZFR1**	RQQKLDT	LSQTLKR	RMDHLAG	9
**hPGRN ZFR2**	RQQKLDT	LSQTLKR	RVDHLGG	10
**hPGRN ZFR3**	RRSRLDV	LSQTLKR	RMDHLAG	3
**hPGRN ZFR4**	RRSRLDV	LSQTLKR	RVDHLGG	3
**hPGRN ZFR5**	TTTKLAI	LSQTLKR	RMDHLAG	5
**hPGRN ZFR6**	TTTKLAI	LSQTLKR	RVDHLGG	5
**hPGRN ZFR7**	TTTKLAI	LSQTLNR	RVDHLGG	5

Binding affinities are expressed as mean fold-activation of transcription in the EZSS system.

**Table 3:** The relative luciferase units revealed by EZSS for hPGRN ZFPs with 1 X TSF, 7 X TSF and the ratios.

ZF name	1X	7X	7x/1x
**ZFL1**	4.0	218.2	54.3
**ZFL2**	1.9	86.8	45.2
**ZFL4**	3.2	160.8	49.6
**ZFR1**	0.6	5.2	8.4
**ZFR2**	0.7	6.6	9.5
**ZFR4**	0.4	0.8	2.0

### Targeted Chromosomal Gene Repair by Heterodimeric ZFNs

**Figure 3:**
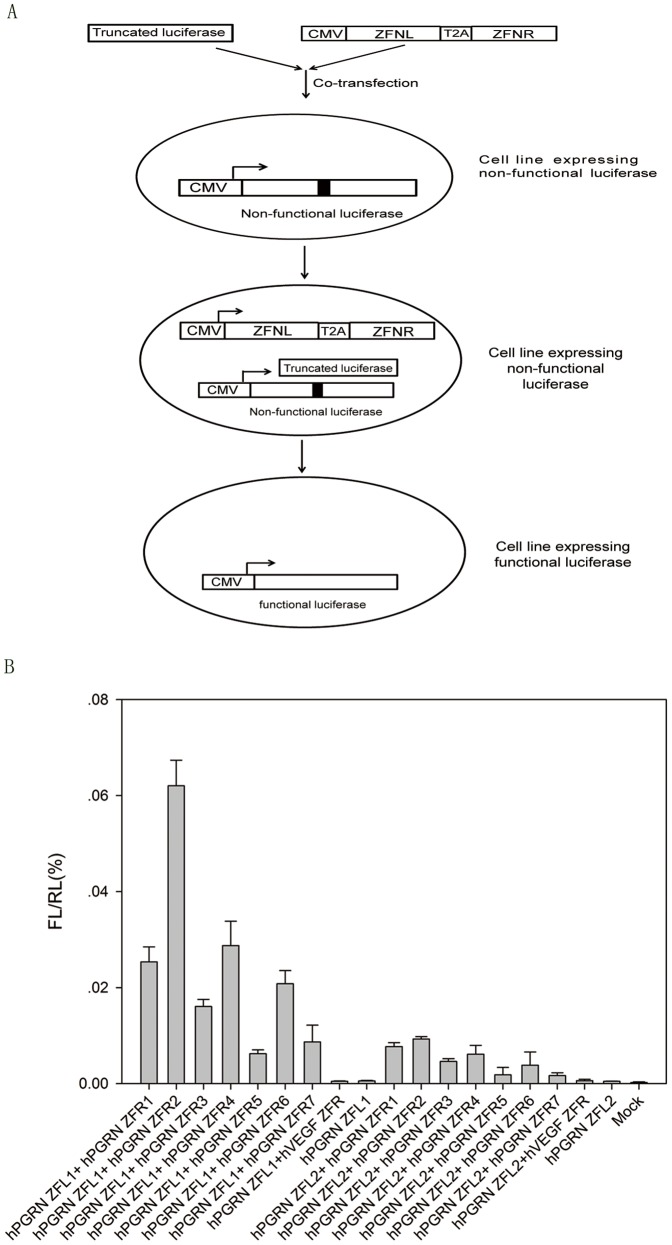
Gene repair assay. (A) Illustration of luciferase restoration in mammalian cells by ZFNs. A stable cell line, which expresses a single copy of mutant firefly luciferase with a TSF for hPGRN ZFN inserted in the central region, was established by knock-in technique mediated by AAVS1 ZFN. Functional firefly luciferase can be restored by co-transfection of hPGRN ZFN and truncated firefly luciferase fragment into the stable cell line. (B) The restored luciferase activities resulting from the transfection of hPGRN ZFNs and donor into the stable cell line expressing a mutant luciferase. Renilla luciferase was transfected as an internal inference. The ratio of firefly luciferase and renilla luciferase activity (FL/RL) was used to reflect the correction efficiency.

Our preliminary data suggested that a ‘failed’ half ZFN might contain enough binding activity to form a working ZFN pair with another binding active half ZFN. To test this hypothesis and to assess whether the assembled ZFN pairs were able to promote gene repair in a chromosome context, we modified a previously described mammalian cell-based GFP (Green Fluorescent Protein, GFP) gene targeting reporter system by replacing single GFP reporter with a mutated firefly luciferase gene with a TSF inserted in the middle [24]. Co-transfection of a truncated firefly luciferase and an effective ZFN could convert the single mutant luciferase gene into the wild-type functional version (Figure 3A). Heterodimeric hPGRN ZFN pairs were assembled by using different hPGRN ZFLs (e.g., ZFL1 and ZFL2) and ZFRs (ZFR 1 to 7). The gene repair efficiency of each hPGRN ZFN was illustrated by luciferase activity (Figure 3B and Table S1). The results showed that the gene repair ability of each hPGRN ZFN pair was in proportion with the combination of the binding activities of the ZFL and ZFR, except small deviations in pairs involving ZFR4 and ZFR5 (Figure S1). Assembled heterodimeric hVEGF2 ZFN pairs also showed similar results. The gene repair efficiency of hPGRN-ZFL1/ZFR2, hPGRN-ZFL1/ZFR4, or hPGRN- ZFL1/ZFR7 was about 85%, 38% or 12% of that of hVEGF1 ZFN.

### Confirm Site-specific Genome Editing by hPGRN ZFNs by Sequencing

**Figure 4:**
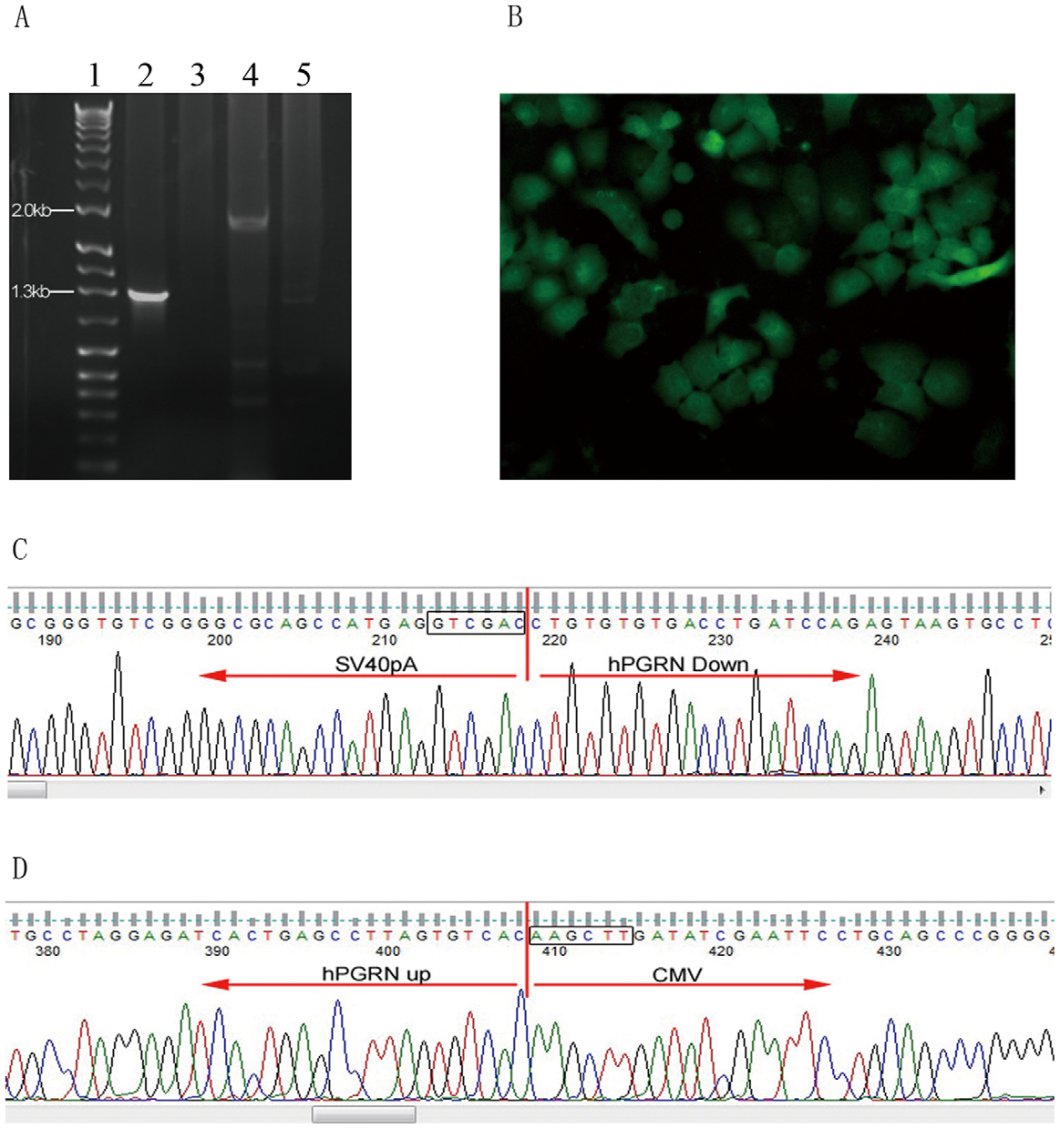
hPGRN ZFN mediated site-specific genomic integration in mammalian genome. (A) The PCR analysis of site-specific integration of exogenous expression cassette into the genome of human PGRN in HepG2 cells. Lane1: DNA marker; Lane2: the fragment amplified from the cells treated by hPGRN ZFN plus donor by using two pairs of primers: one located downstream of the down arm in the genome, the other located in exogenous expression cassette; Lane3: the fragment amplified from the cells treated by donor only by using similar primers to Lane2; Lane4: the fragment amplified from the cells treated by hPGRN ZFN plus donor by using two pairs of primers: one located upstream of the up arm in the genome, the other located in exogenous expression cassette; Lane5: the fragment amplified from the cells treated by donor only by using similar primers to Lane 4. (B) eGFP expression by the expression cassette integrated in the genome of HepG2 cell line. HepG2 cells were screened and cloned in the presence of neomycin and GCV post co-transfection of hPGRN ZFN and the donor vector pAd5-E1-hPGRN-CMV-eGFP-T2A-neo-up/down-PGK-TK-T2A-mcherry. (C) The sequence of the region containing down arm of the homologous fragment and 3 terminus of the exogenous expression cassette. (D) The sequence of the region containing up arm of the homologous fragment and 5 terminus of the exogenous expression cassette.

**Figure 5:**
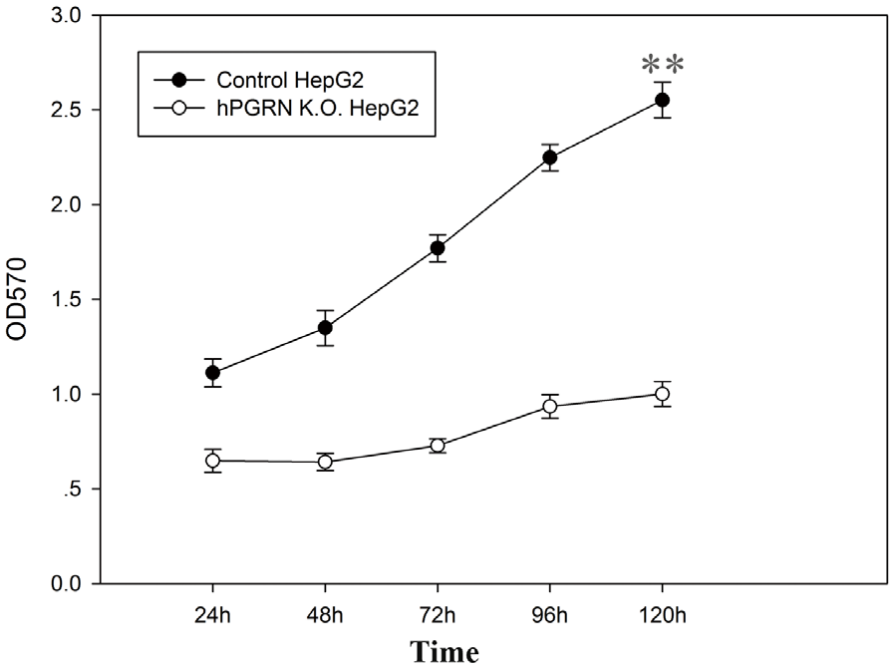
Decreased cell growth of PGRN knock-out cell line as determined by the MTT assay. The control HepG2 or PGRN K.O. HepG2 cells were plated at 5,000 cells/well in 96-well plates with 200 µl DMEM medium and incubated in a humidified atmosphere containing 5% CO2 at 37°C. Then 20 µl 3-(4, 5-dimethylthiazol-2-yl)-2,5-diphenyltetrazolium bromide (MTT) stock solution (5 mg/ml) was added to each well at different time points. After 4 h of incubation, the medium was removed carefully and the converted dye was solubilized with 200 µl DMSO. The absorbance of the converted dye was measured at a wavelength of 570 nm. The data are expressed as mean ± SD of three independent experiments. **P<0.001.

In order to confirm site-specific genome editing mediated by hPGRN ZFNs by sequencing, a donor vector containing an CMV-eGFP-T2A-neomycin expression cassette flanked by homologous arms and a PGK-TK-T2A-mCherry-pA expression cassette located outside of the homologous arms were co-transfected with pAd5-E1-hPGRN-ZFN (hPGRN-ZFL1/ZFR2) (Table S2) into HepG2 cells followed with positive (G418) and negative (GCV) selection. Positive G418 selection enabled us to recover the cells with targeted integration or random integration that carried the exogenous neomycin gene. The following negative GCV selection eliminated those cells surviving the positive selection with random integration that carried TK gene. Targeted donor recombination at the *pgrn* locus was demonstrated by PCR (Figure 4A), eGFP expression (Figure 4B) and sequencing (Figure 4C, D). The PGRN knock-out cell line showed significantly decreased cell growth compared with the control (Figure 5).

## Discussion

In this paper we established a highly sensitive mammalian cell-based transcriptional reporter system to unveil the potential ZFP candidates among those with low binding activities. Our results showed that this system increased the screening sensitivity up to 50-fold and markedly amplified the differences in the binding activities between different ZFPs. We also found that the targeted chromosomal gene repair efficiency of each PGRN ZFN pair was in proportion with the combination of the binding activities of the ZFL and ZFR. A hPGRN ZFR with low binding ability was able to form a biological active ZFN if combined with a hPGRN ZFL with relatively high binding ability. Lastly, site-specific genome editing by hPGRN ZFNs generated by this system was confirmed by sequencing, and the PGRN knock-out cell line showed significantly decreased cell growth compared with the control. Compared with the selection methods, which are highly labor-intensive and time-consuming, modular assembly is faster and easier to engineer ZFPs. Simply by using a computer-generated archive the researchers can build ZFNs from one- or two-finger modules [14], [15], [16], [17], [18]. However, only 24% of the ZFN candidates showed *in vivo* activities [8]. Recently, Isalan proposed a scheme to improve the success rate of modular assembly method [19]. Briefly, when fingers fail, *in vitro* cutting assays can rapidly identify whether one or both halves of the nuclease are working. When both halves fail, choose another target site. When only one half of the nuclease fails, screen for variants with a mini-library by different methods including bacterial one-hybrid [25] and two hybrid systems [15], or yeast one-hybrid system [19], [20] to rescue ‘orphan’ half-ZFNs.

In this paper, ZFPs assembled based on the publicly available resources for single zinc finger modules were screened for binding activities by using a mammalian cell-based reporter system, followed by analysis of genome editing activities. The results suggested that ZFNs with good genome editing activities did not necessarily have high binding abilities on both right and left ZFPs. For example, a right hPGRN ZFP with low binding ability was able to form a biological active hPGRN ZFN if combined with a left hPGRN ZFP with relatively high binding ability. The targeted chromosomal gene repair efficiency of each hPGRN or hVEGF2 ZFN pair was in proportion with the combination of the binding activities of the ZFL and ZFR. Consistent with our observation, an analysis of off-target cutting also suggested that a shortage of binding energy from a half-site can be energetically compensated by excess zinc finger: DNA binding energy in the other half-site [21].

Most of ZFNs generated in this study were shown to have site-specific genome editing abilities in mammalian genome. The overall high success rate of our system was partially due to the highly sensitive screening for ZFP candidates with binding activities by our mammalian cell-based luciferase reporter system. Our results showed that with insertion of multiple copies of ZFN TSF of an interested gene, this system increased the screening sensitivity up to 50-foldand markedly amplified the differences in the binding activities between different ZFPs, which allowed us to unveil the potential ZFP candidates among those with low binding activities. In addition to high sensitivity, this system is also simple and easy to utilize. However, there were still rare ZFPs with relatively high binding ability but low targeted genome editing activity. The underlying mechanism may be related to chromatin accessibility or the 3-D structure of ZFPs. There is a need for further study.

Previous studies suggested that ZFN-associated cytotoxicity was due to the creation of off-target DSBs [26], [27], [28], which can be detected by immunofluorescent staining of 53BP1 [29], [30], [31], [32]. Consistent with the observation of Pattanayak et al. [21], our data showed that ZFN pairs with high binding activities were inclined to have higher cytotoxicities (Data not shown). The generation of a ZFN with balanced DNA-binding activity and toxicity is critical for some applications, and our system can be a platform for further optimizing the nucleases with regard to specificity and cytotoxicity.

## Materials and Methods

### Cell Culture

Human kidney cell line, HEK 293 and human hepatocyte cell line, HepG2 were purchased from ATCC, cultured in 10% DMEM (high glucose) (Invitrogen, Carlsbad, CA) containing 10% newborn calf serum. Cell lines with a site specific integration of exogenous genes in the genome were positively or negatively screened in the presence of selection medium containing G418 (200 µg/ml) or GCV (0.5–1.0 mg/ml). Selection medium was changed every two days.

### Assembling and Screening of Zinc Finger Protein

Zinc finger arrays were selected using the OPEN method essentially as previously described [15]. To create recombinant libraries of zinc finger arrays for use in selections, zinc finger pools for targeting triplet subsites were amplified by PCR using a synthetic biologically active human VEGF ZFP [15] as a template (called hVEGF1 ZFP). Amplified finger pool products were purified on 10% polyacrylamide gels and then fused together by overlap PCR to create three-finger arrays. A total of six primers were used to construct each ZFP. All ZFPs we designed have an identical frame. We successfully produced 7 new ZFPs for human VEGF genome (named hVEGF2 ZFP) with 4 of them targeting the left and the other 3 targeting the right region of the target sequence (Table 1). Similarly, we produced 11 ZFPs for human progranulin (PGRN) genome (4 for the left and 7 for the right region) (Table 2). The system for screening assembled ZFP was developed by (1) the construction of transcription factor consisting of p65 transcription activation domain ranging from 288 aa to 548 aa of p65 protein located at C-terminal and left zinc finger protein fused with nuclear localization signal and flag tag or right zinc finger protein fused with nuclear localization signal and HA tag located at N-terminal; (2) the generation of reporter vector by inserting different copy number of TSF recognized by assembled ZFP into pGL3 vector (Promega, Madison, WI), a mini-CMV promoter was introduced downstream of TSF. Assembled ZFPs were screened based on the firefly luciferase activity by co-transfecting two vectors above into HEK 293 cell. Renilla luciferase was transfected as internal reference.

### Construction of ZFN Expressing Plasmid and Donor Vector

Left zinc finger nuclease (LZFN) containing zinc finger protein (ZFP) targeted human VEGF and mutated Fok1 [33] was cloned into adenoviral E1 shuttle by ClaI and XbaI site, the resultant plasmid was named pAd5 E1-CMV-hVEGF1-LZFN. And right zinc finger nuclease (RZFN) containing zinc finger protein targeted human VEGF and mutated Fok1 [33] was cloned into adenoviral E1 shuttle by EcoRI and SpeI site, the obtained plasmid was called pAd5 E1-CMV-hVEGF1-RZFN. The sequence of hVEGF1 ZFN targeting human genome was based on previously published data (VF2468) [15]. Flag and HA tag were respectively introduced at N-terminal of left and right ZFP. A fusion protein containing left and right ZFN isolated by T2A ‘self-cleaving’ peptide was cloned into an adenoviral E1 shuttle by ClaI and SpeI sites, which is under the control of CMV promoter, the obtained plasmid was called pAd5 E1-hVEGF1-L/R-ZFN. Using similar strategy, we obtained pAd5E1-hVEGF2-L/R-ZFN containing a pair of new ZFNs screened targeting human VEGF based on the method developed above, which recognizes new target sequence present in human VEGF genome, pAd5 E1-hPGRN-L/R-ZFN containing human ZFNs screened recognizing target sequence present in the human PGRN genome.

Donor DNA vector was constructed by a sequential cloning of up and down homologous sequence targeting human PGRN amplified from human genomic DNA respectively, into Ad5 E1 shuttle. The cassette expressing eGFP-T2A-neo under the control of CMV promoter was inserted into donor DNA vector between up and down homologous sequence, and the cassette expressing TK-T2A-mcherry under the control of PGK promoter was inserted downstream of the down homologous sequence. The resultant vector was called pAd5-E1-hPGRN-CMV-eGFP-T2A-neo-up/down-PGK-TK-T2A-mcherry, which could be used for positive and negative selection (All primer sequences used for the vector construction will be provided upon request).

### Site specific Integration of a Foreign DNA Sequence in to PGRN Genome Mediated by ZFNs

Donor vector pAd5-E1-hPGRN-CMV-eGFP-T2A-neo-up/down-PGK-TK-T2A-mcherry was co-transfected with pAd5-E1-hPGRN-L/R-ZFN into HepG2, the cells were then cultured in the presence of neomycin 48 hour post-transfection, followed by the screening of GCV. The cell line with site specific integration in the genome of HepG2 was used for extracting genomic DNA. Site specific integration was confirmed by the sequence of amplified fragment using indicated primers based on the template of genomic DNA extracted from cells above. (All primer sequences will be provided upon request).

### Establishment of the Cell Lines for the Analysis of Biological Activity of Assembled ZFNs

ZFPs targeting AAVS1 were synthesized based on previously published data [34]. Synthesized right and left ZFP as NH2-terminal were fused with catalytic domain of FokI, respectively [33]. Flag and HA tag were respectively introduced at N-terminal of left and right ZFP. Left and right ZFN was isolated by T2A ‘self-cleaving’ peptide under the control of CMV promoter, the obtained plasmid was called pAd5 E3-AAVS1-L/R-ZFN. To generate donor vector for the knock-in of mutant firefly luciferase gene, a 700 bp N terminal fragment of full length firefly luciferase with a stop codon in the 3′ end and a 1000 bp C terminal fragment of full length firefly luciferase was isolated by TSF recognized by hPGRN ZFN. The resultant mutant firefly luciferase was under control of CMV promoter. The plasmid was called pAd5E1-CMV-luciferase-Mu. Neomycin expression cassette under the control of EF1a promoter was located at the 3 terminal of mutant firefly luciferase expression cassette. The obtained plasmid was called pAdE1-CMV-luciferase-mu-EF1a-neomycin. The AAVS1 up and down arm was respectively cloned at 5 terminal of mutant firefly luciferase expression cassette and 3 terminal of neomycin expression cassette. The resultant plasmid was named pAd5-E1-AAVS1-Up-CMV-luciferase-mu-EF1a-neomycin-Down. The expression cassette of TK and mCherry reporter gene isolated by T2A ‘self-cleaving’ peptide under the control of PGK promoter were inserted at the 3 terminal of AAVS1 down arm as negative selection marker. The final donor plasmid was called pAdE1-AAVS1-Up-CMV-luciferase-mu-EF1a-neomycin- Down-PGK-TK-T2A-mCherry. To build 293-luciferase-hPGRN-TSF cell line, HEK 293 cells were transfected with AAVS1 ZFN expression vector and donor vector at an efficiency of ∼80% using Lipofectamine 2000 Reagent (Invitrogen) in Opti-MEMI reduced serum medium according to the manufacturer’s protocol and then screened and cloned in the presence of the medium containing neomycin and GCV.

### Luciferase Assay

ZFP-p65 AD expressing plasmid and renilla luciferase expressing plasmid as an internal control were co-transfected with reporter vector containing a different copy number of TSF into HEK 293 cells. Renilla luciferase expressing plasmid and reporter vector were co-transfected into HEK 293 cells as negative control. 48 hours post-transfection, the cell lysates were subject to luciferase activity assay using Dual Luciferase Assay kit (Promega, Madison, WI, E1910) according to the manufacture manual using Thermo Scientific Varioskan Flash (Thermo, Waltham, MA).

### Western Blot

ZFNs expressing plasmid was transfected into HEK 293 cells, 48 hours post-transfection, the cell lysates were subject to SDS-PAGE, then transferred on N.C membrane, followed by the incubation with McAb against HA or Flag. The film was developed using ECL kit (Thermo) according to manufacture manual.

## Supporting Information

Figure S1 **Scatter plot of binding abilities (Y) against gene repair activities (X) of hPGRN ZFNs.** The gene repair ability of each hPGRN ZFN pair was in proportion with the combination of the binding activities of hPGRN ZFL1 and each paired ZFR (i.e., ZFR1 to ZFR7) except small deviations in pairs involving ZFR4 and ZFR5 (see arrows).
(TIF)

Table S1 **Raw data of luciferase correction assay.** The biological activities of hPGRN ZFNs were expressed as firefly luciferase values normalized to renilla luciferase (FL/RL).
(DOC)

Table S2 **Full DNA and protein sequences of the best ZFN (hPGRN ZFL1 and ZFR2) for human PGRN.**
(DOC)
