# Supplementary figures and images for: Rescue the Failed Half-ZFN by a Sensitive Mammalian Cell-Based Luciferase Reporter System
Source: PLoS One. 2012 Sep 18;7(9):e45169. doi: 10.1371/journal.pone.0045169 (PMC3445457; doi:10.1371/journal.pone.0045169)

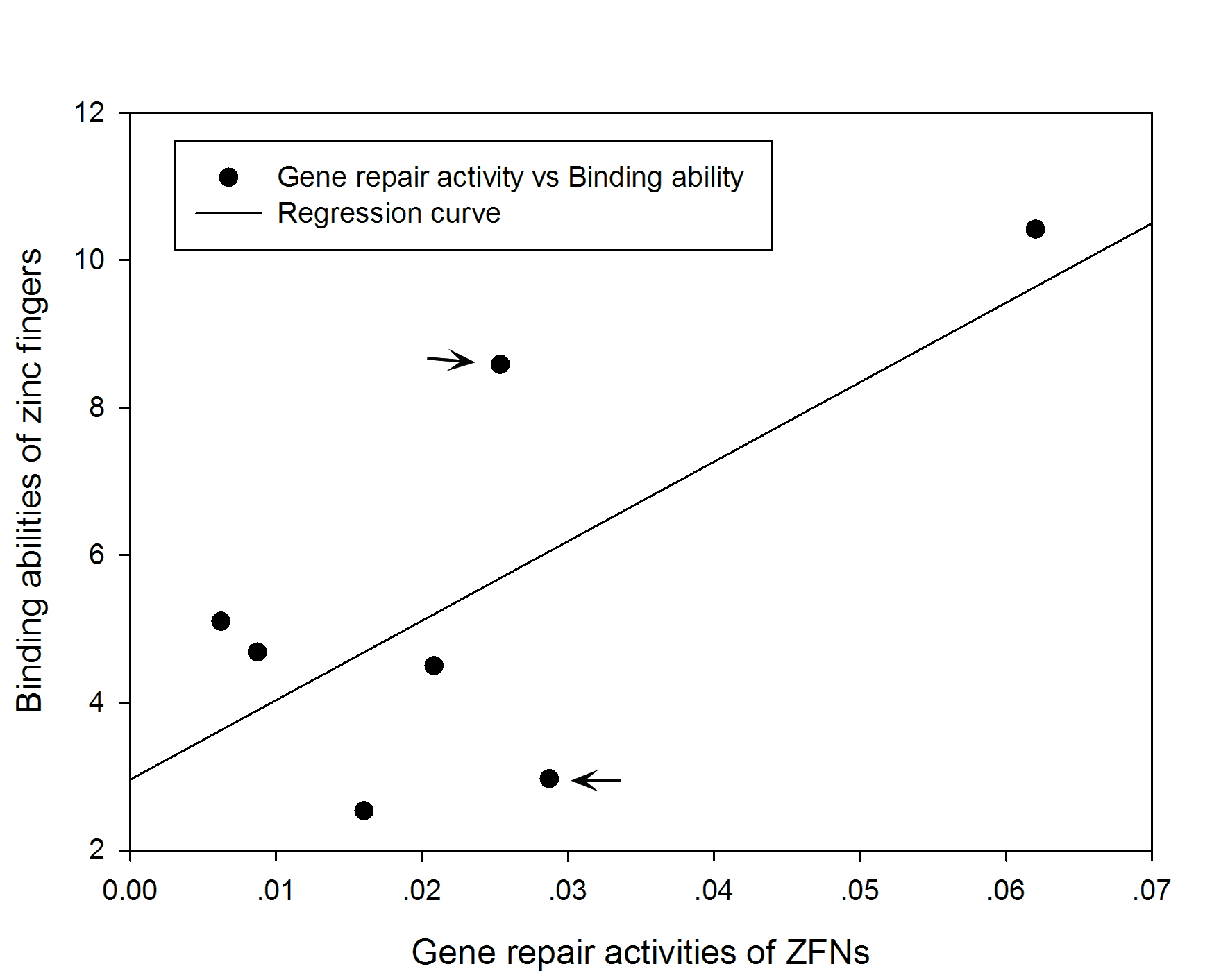

Supplement: Figure S1 — Scatter plot of binding abilities (Y) against gene repair activities (X) of hPGRN ZFNs. The gene repair ability of each hPGRN ZFN pair was in proportion with the combination of the binding activities of hPGRN ZFL1 and each paired ZFR (i.e., ZFR1 to ZFR7) except small deviations in pairs involving ZFR4 and ZFR5 (see arrows). (TIF) [file pone.0045169.s001.tif]
