# Supplementary material for: Rescue the Failed Half-ZFN by a Sensitive Mammalian Cell-Based Luciferase Reporter System
Source: PLoS One. 2012 Sep 18;7(9):e45169. doi: 10.1371/journal.pone.0045169 (PMC3445457; doi:10.1371/journal.pone.0045169)
Supplement: Table S1 — Raw data of luciferase correction assay. The biological activities of hPGRN ZFNs were expressed as firefly luciferase values normalized to renilla luciferase (FL/RL). (DOC) [file pone.0045169.s002.doc]

**Supplemental Table 1. Raw data of luciferase correction assay.** The biological activities of hPGRN ZFNs were expressed as firefly luciferase values normalized to Renilla luciferase (FL/RL).

| **ZF Name** | **Mean value of FL/RL (%)** | **S.D. of FL/RL** |
| --- | --- | --- |
| **hPGRN ZFL1+ hPGRN ZFR1** | 0.0253667 | 0.003092 |
| **hPGRN ZFL1+ hPGRN ZFR2** | 0.0620333 | 0.005348 |
| **hPGRN ZFL1+ hPGRN ZFR3** | 0.0160333 | 0.001457 |
| **hPGRN ZFL1+ hPGRN ZFR4** | 0.0287333 | 0.005103 |
| **hPGRN ZFL1+ hPGRN ZFR5** | 0.0062409 | 0.000787 |
| **hPGRN ZFL1+ hPGRN ZFR6** | 0.0208333 | 0.002701 |
| **hPGRN ZFL1+ hPGRN ZFR7** | 0.0087265 | 0.003489 |
| **hPGRN ZFL1+ hVEGF1 ZFR** | 0.0004957 | 8.27E-05 |
| **hPGRN ZFL1** | 0.000554 | 0.000132 |
| **hPGRN ZFL2+ hPGRN ZFR1** | 0.0077322 | 0.000813 |
| **hPGRN ZFL2+ hPGRN ZFR2** | 0.009319 | 0.000473 |
| **hPGRN ZFL2+ hPGRN ZFR3** | 0.0046253 | 0.00057 |
| **hPGRN ZFL2+ hPGRN ZFR4** | 0.0061328 | 0.00185 |
| **hPGRN ZFL2+ hPGRN ZFR5** | 0.0018476 | 0.001521 |
| **hPGRN ZFL2+ hPGRN ZFR6** | 0.0038495 | 0.002726 |
| **hPGRN ZFL2+ hPGRN ZFR7** | 0.001688 | 0.000573 |
| **hPGRN ZFL2+ hVEGF1 ZFR** | 0.000626 | 0.000255 |
| **hPGRN ZFL2** | 0.0004905 | 4.88E-05 |
| **Mock** | 0.0002471 | 0.00012 |
