# Supplementary material for: Rescue the Failed Half-ZFN by a Sensitive Mammalian Cell-Based Luciferase Reporter System
Source: PLoS One. 2012 Sep 18;7(9):e45169. doi: 10.1371/journal.pone.0045169 (PMC3445457; doi:10.1371/journal.pone.0045169)
Supplement: Table S2 — Full DNA and protein sequences of the best ZFN (hPGRN ZFL1 and ZFR2) for human PGRN. (DOC) [file pone.0045169.s003.doc]

**Supplemental Table 2. Full DNA and protein sequences of the best ZFN (hPGRN ZFL1 and ZFR2) for human PGRN.**

1. **DNA sequence of hPGRN ZFL1-FokI DD and ZFR2-FokI RR:**

hPGRN ZFL1-FokI DD:

ATGGCACCAAAGAAAAAGCGGAAGGTAGATTACAAAGATCATGATGGCGATTACAAGGACCACGATATCGACTACAAAGATGACGATGATAAGAAGCTTCCCCACGAGAGACCTTTCCAGTGCAGGATCTGTATGCGGAACTTCAGCGACAGAAGCAACCTGACCAGGCACACAAGGACCCATACTGGCGAAAAGCCCTTTCAATGTCGGATTTGCATGCGCAACTTTTCCCTGAGACACCATCTCACAAGACACCTCCGGACACACACCGGAGAGAAACCATTCCAGTGTAGAATCTGCATGAGGAATTTCTCTCAGAAGGCCAACCTGACCAGGCATCTGAGAACCCATCTCGAGGGCGGTGGCAGCCAGCTGGTTAAATCCGAGTTGGAAGAGAAAAAGTCTGAGCTCCGCCATAAGTTGAAATACGTGCCTCACGAGTATATCGAACTGATCGAGATCGCCAGAAACTCAACCCAAGACAGGATTTTGGAAATGAAAGTGATGGAGTTCTTTATGAAGGTCTATGGCTATAGGGGAAAGCACCTCGGCGGGAGCAGGAAGCCCGACGGCGCCATTTATACAGTCGGGTCTCCAATCGACTATGGGGTCATCGTTGACACTAAGGCCTATTCCGGGGGTTACAACCTCCCAATAGGGCAGGCTGACGAGATGCAGGACTACGTGGAGGAGAACCAAACAAGGAACAAGCATATAAACCCTAACGAGTGGTGGAAAGTATACCCTAGTTCTGTTACTGAGTTCAAGTTTCTCTTCGTGAGCGGACACTTCAAAGGAAATTACAAAGCTCAACTGACAAGACTGAATCATATTACTAACTGTAATGGTGCCGTCCTGTCAGTGGAGGAACTGCTGATTGGCGGAGAGATGATCAAGGCAGGCACCCTTACTCTCGAAGAAGTGCGGCGAAAGTTTAATAACGGTGAAATCAACTTCTAA

hPGRN ZFR2-FokI RR:

ATGGCGCCCAAGAAGAAACGAAAGGTCTATCCTTACGATGTGCCAGACTACGCCGGGTATCCATATGATGTGCCTGACTATGCCGGCAGCTATCCCTATGACGTGCCCGATTATGCAGCTCACGGTACCCCCCACGAGAGACCTTTCCAGTGCAGGATCTGTATGCGGAACTTCAGCCGCCAACAGAAGCTGGACACCCACACAAGGACCCATACTGGCGAAAAGCCCTTTCAATGTCGGATTTGCATGCGCAACTTTTCCCTCAGCCAGACACTGAAGAGGCACCTCCGGACACACACCGGAGAGAAACCATTCCAGTGTAGAATCTGCATGAGGAATTTCTCTCGCGTGGACCACCTGGGGGGCCATCTGAGAACCCATGGATCCGGCGGTGGCAGCCAGTTGGTGAAATCCGAGCTGGAGGAGAAAAAGTCAGAGCTGCGCCACAAACTCAAGTACGTGCCACACGAATACATTGAGCTGATCGAGATCGCCAGGAACTCCACGCAGGACAGAATCCTGGAGATGAAGGTAATGGAATTTTTCATGAAGGTGTACGGCTACAGAGGCAAGCATCTGGGAGGGTCCCGCAAGCCTGATGGAGCAATCTACACCGTCGGAAGCCCCATAGATTACGGGGTAATCGTCGATACCAAAGCATATAGTGGCGGATACAACCTGCCAATCGGCCAAGCCCGGGAAATGCAGCGATACGTGGAAGAAAACCAGACTAGGAACAAACACATTAACCCAAACGAATGGTGGAAAGTCTATCCTAGCTCTGTGACGGAGTTCAAGTTTCTCTTTGTTTCCGGCCATTTCAAGGGGAATTACAAGGCTCAGCTGACAAGGCTGAATCATATTACTAATTGTAACGGGGCCGTTCTCTCAGTGGAAGAGCTGCTGATTGGCGGAGAGATGATTAAAGCCGGCACCCTTACCCTGGAAGAGGTTCGGCGGAAATTCAACAATGGCGAGATAAACTTTTGA

**2. Amino acid sequence of hPGRN ZFL1-FokI DD and ZFR2-FokI RR:**

hPGRN ZFL1-FokI DD:

MAPKKKRKVDYKDHDGDYKDHDIDYKDDDDKKLPHERPFQCRICMRNFSDRSNLTRHTRTHTGEKPFQCRICMRNFSLRHHLTRHLRTHTGEKPFQCRICMRNFSQKANLTRHLRTHLEGGGSQLVKSELEEKKSELRHKLKYVPHEYIELIEIARNSTQDRILEMKVMEFFMKVYGYRGKHLGGSRKPDGAIYTVGSPIDYGVIVDTKAYSGGYNLPIGQADEMQDYVEENQTRNKHINPNEWWKVYPSSVTEFKFLFVSGHFKGNYKAQLTRLNHITNCNGAVLSVEELLIGGEMIKAGTLTLEEVRRKFNNGEINF

hPGRN ZFR2-FokI RR:

MAPKKKRKVYPYDVPDYAGYPYDVPDYAGSYPYDVPDYAAHGTPHERPFQCRICMRNFSRQQKLDTHTRTHTGEKPFQCRICMRNFSLSQTLKRHLRTHTGEKPFQCRICMRNFSRVDHLGGHLRTHGSGGGSQLVKSELEEKKSELRHKLKYVPHEYIELIEIARNSTQDRILEMKVMEFFMKVYGYRGKHLGGSRKPDGAIYTVGSPIDYGVIVDTKAYSGGYNLPIGQAREMQRYVEENQTRNKHINPNEWWKVYPSSVTEFKFLFVSGHFKGNYKAQLTRLNHITNCNGAVLSVEELLIGGEMIKAGTLTLEEVRRKFNNGEINF
